# Diversity of culture-independent bacteria and antimicrobial activity of culturable endophytic bacteria isolated from different *Dendrobium* stems

**DOI:** 10.1038/s41598-019-46863-9

**Published:** 2019-07-17

**Authors:** Shan-Shan Wang, Jia-Meng Liu, Jing Sun, Yu-Feng Sun, Jia-Ni Liu, Ning Jia, Bei Fan, Xiao-Feng Dai

**Affiliations:** 1 Institute of Food Science and Technology, Chinese Academy of Agricultural Sciences, Beijing, China; 2 Key Laboratory of Agro-products Quality and Safety Control in Storage and Transport Process, Ministry of Agriculture and Rural Affairs, Beijing, China

**Keywords:** Applied microbiology, Bacterial host response

## Abstract

*Dendrobium* is known for its pharmacological actions including anti-cancer effect, anti-fatigue effect, gastric ulcer protective effect, and so on. At present, only studies on endophytic fungi of *Dendrobium* affecting the metabolites of host plants have been reported, very little research has been done on endophytic bacteria. In this study, we have demonstrated the great diversity of endophytic bacteria in 6 *Dendrobium* samples from different origins and cultivars. According to the results of the culture-independent method, the endophytic bacterial community in *Dendrobium* stems showed obvious different in the 6 samples and was influenced by origin and cultivar. Some bacteria including *Ralstonia*, *Comamonas* and *Lelliottia* were first detected in *Dendrobium* in this study. Based on the culture-dependent method, a total of 165 cultivable endophytic bacteria isolates were isolated from the sterilized *Dendrobium* stems, and were classified into 43 species according to the 16S rRNA gene sequence analysis. Moreover, 14 of the 43 strains showed antimicrobial activity against phytopathogen using the Kirby-Bauer method. Strain NA-HTong-7 (*Bacillus megaterium*, 99.12%) showed the highest antimicrobial activity. This study was the first comprehensive study on endophytic bacteria of *Dendrobium* from different origins and cultivars, which provides new insights into the endophytic bacteria from *Dendrobium*.

## Introduction

*Dendrobium* is one of the largest genera among *Orchidaceae* family plants about 1500 species distributed all over the world. There are 74 species and 2 variants of genus *Dendrobium* Sw. in China, mainly distributed in south and southwest of China^1^. *Dendrobium*, as the Traditional Chinese Medicine (TCM), makes tremendous contributions to public healthcare and promotes the development of TCM^2,3^. According to the Chinese pharmacopoeia, *Dendrobium* has great clinical effect on human stomach and kidney^4^. Modern pharmacological actions of *Dendrobium* in hepatoprotective effect, anti-cancer effect, hypoglycemic effect, anti-fatigue effect, gastric ulcer protective effect, and so on were also reported. This may mainly be attributed to polysaccharides which are considered as the major bioactive components of *Dendrobium*^5^.

Endophytes are a class of endosymbiotic microorganisms widespread among plants that colonize intercellular and intracellular spaces of all known plant compartments but do not cause any plant diseases or significant morphological changes^6^. The endophyte is likely to be an important determinant of plant health and productivity, and has received substantial attention in recent years as a subject of scientific and commercial interest^7^. Numerous studies have highlighted the ability of plant-associated microbes to influence important traits such as growth, disease resistance, abiotic stress tolerance, water retention, and the synthesis of plant growth-promoting hormones^8,9^. They produce a large amount of novel and bioactive secondary metabolites that are not only beneficial to the host plants but also economically important to humans for the potential applications in pharmaceutical, agricultural, and food industries^10,11^. In view of the importance of endophytes to plants metabolites, recent studies have begun to probe the importance of endophytes to medicinal plants^12–15^. Studies have shown that the content of secondary metabolites from the same medicinal plant species can be different depending on their location of cultivation, which could in part be related to different composition in their associated microbes when grown at different sites.

At present, only studies on endophytic fungi of *Dendrobium* have been reported, very little research has been done on endophytic bacteria. In the published literature, most of the studies on endophytes of *Dendrobium* were mainly based on the culture-dependent method^16–20^. However, less than 1% of bacteria can be usually be cultured, which is detrimental to the study of the comprehensive roles of endophytic bacteria^21^. Therefore, in this study, Illumina MiSeq was used to sequence the V5–V7 regions of the bacteria 16S rRNA gene, and combined with the culture-dependent methods to identify the endophytic bacteria in the *Dendrobium* samples. At the same time, the Kirby-Bauer test was used to screen for antimicrobial activity of the isolated endophytic bacterial strain. The results will provide new insights into the endophytic bacteria from *Dendrobium*.

## Results

### Diversity of endophytic bacteria based on Illumina-based analysis

#### Quality control and analysis of sequencing data

A total of 713, 422 reads and 655 OTUs were obtained after clustering at a 97% similarity level from 6 samples (Three parallel in each sample). Each library contained 29, 709 to 54, 747 reads. The rarefaction curves showed that the sequencing work was relatively comprehensive in covering the bacterial diversity, as the curves tended to approach saturation, indicating that the selected sequence data adequately reflected the bacterial abundance of these samples. In other words, the sequencing data covered almost all the species in the community of the samples under the indication of the rarefaction curve (Supplementary Fig. S1). On the other hand, the distributions of endophytic microbial composition in different *Dendrobium* stems were detected on different taxonomy level and other bacterial diversity indices including Shannon, Simpson, Ace and Chao were applied for the estimating of endophytic community complexity. All library coverage is above 99.97% (Supplementary Table S1).

#### Composition and diversity of the endophytic bacterial community in *Dendrobium* samples

As many as 99.99% of the bacterial sequences were classified from phylum to genus according to the program QIIME using the default setting, while 0.01% of bacterial sequences were unclassified. The valid sequences were classified into 22 different phyla, 40 classes, 93 orders, 190 families, and 373 genera. The overall bacterial composition of each sample was similar, while the distribution was varied. *Proteobacteria* (55.24%) was the dominant phylum, following by *Actinobacteria* (25.58%), *Firmicutes* (12.86%) and *Bacteroidetes* (5.46%). While the remaining 0.86% contained eighteen very low-abundant phyla (Supplementary Fig. S2).

**Figure 1:**
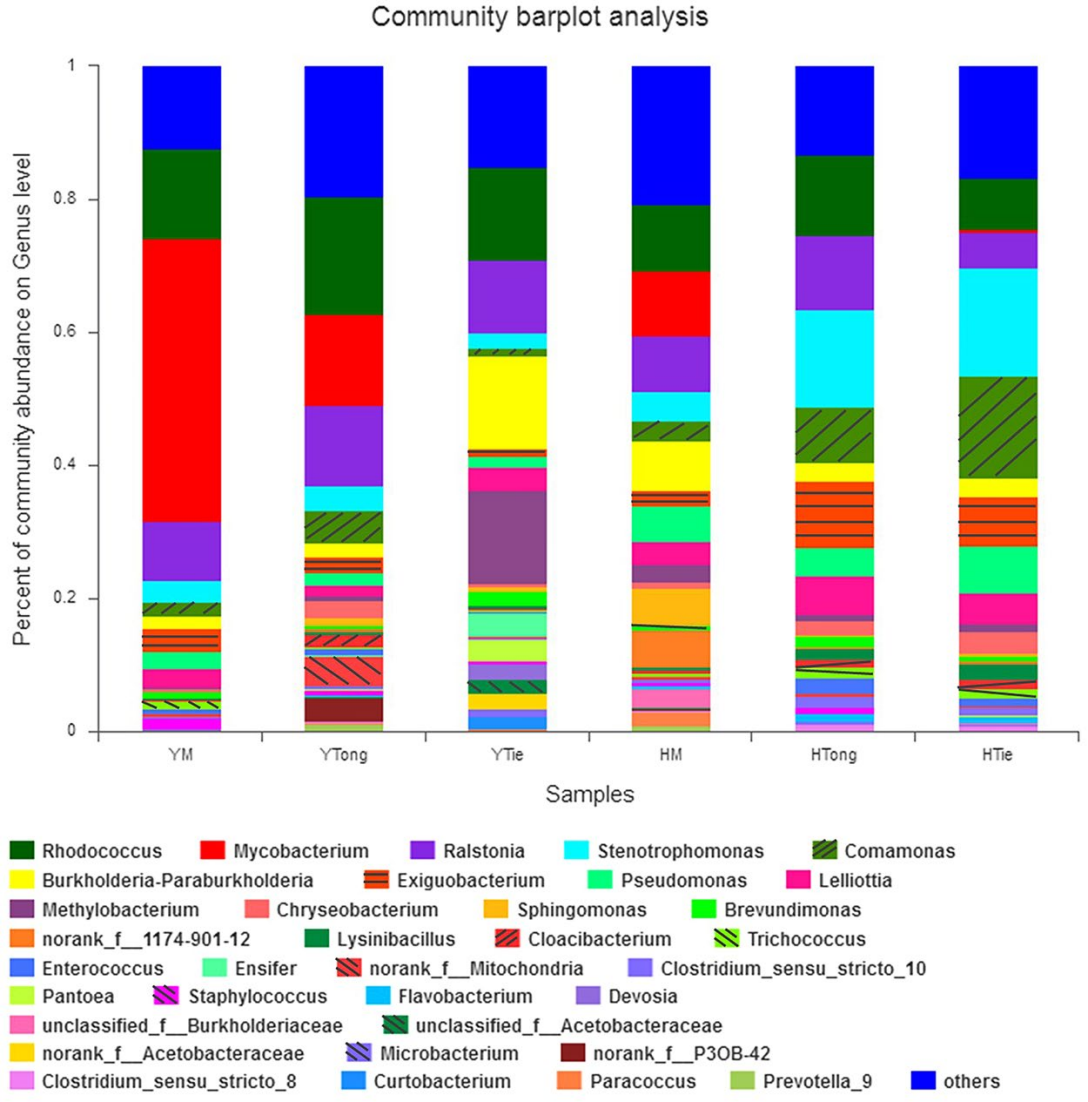
Composition and relative abundance of endophytic bacterial in different samples on the genus level (The color of the column represents the different genera, and the length of the column represents the proportion size of the genus. Sequences that could not be classified into any known group were assigned as “unclassified”. Genera making up less than 1% of total composition in each sample were classified as “other”).

The main endophytic bacterial community for each sample was also analyzed at the genus level (Fig. 1). The results showed that the distribution of each dominant genus among the 6 *Dendrobium* samples was varied.

On the whole, the TiePi samples (YTie and HTie) have higher community abundance than other samples but irrelevant to the origins, followed by the TongPi (YTong and HTong) samples, and the MiHu (YM and HM) samples have lowest community abundance. For every single sample, the dominant genera were different from each other (Table 1). For YM, there were 15 genera with over 1% abundance, and the dominant genera were *Mycobacterium* (42.68%) and *Rhodococcus* (13.44%). For YTie, there were 15 genera with over 1% abundance, and the dominant genera were *Burkholderia-Paraburkholderia* (14.03%), *Methylobacterium* (13.98%), *Rhodococcus* (13.86%) and *Ralstonia* (10.95%). For YTong, there were 13 genera with over 1% abundance, and the dominant genera were *Rhodococcus* (17.60%), *Mycobacterium* (13.74%) and *Ralstonia* (12.04%). For HM, there were 13 genera with over 1% abundance, and the dominant genus was *Rhodococcus* (10.06%). For HTie, there were 17 genera with over 1% abundance, and the dominant genera were *Stenotrophomonas* (16.35%) and *Comamonas* (15.25%). For HTong, there were 15 genera with over 1% abundance, and the dominant genera were *Stenotrophomonas* (14.53%), *Rhodococcus* (12.23%) and *Ralstonia* (11.21%). The relative abundance of the same genus was significant difference among the samples. For example, *Acetobacteraceae*, *Curtobacterium*, *Devosia*, *Ensifer* and *Pantoea* were only detected in YTie sample. *Staphylococcus* and *Sphingomonas* was the specific genus for YM and HM, respectively. Although *Mycobacterium* was detected in all five samples except HTong, but it was only dominant in YM.

**Table 1:** Comparison of percentage (%) of the metagenome sequences affiliated with the dominant bacterial genera (average abundance >1%) for the 6 *Dendrobium* samples.

Genus	YM	YTie	YTong	HM	HTie	HTong
*Acetobacteraceae*	—	4.32	—	—	—	—
*Burkholderia-Paraburkholderia*	1.82	14.03	2.10	7.23	2.82	2.72
*Brevundimonas*	0.90	2.07	—	—	0.91	1.52
*Comamonas*	2.23	1.04	4.80	3.11	15.25	8.52
*Curtobacterium*	—	1.79	—	—	—	—
*Chryseobacterium*	0.47	—	2.61	0.96	3.25	2.14
*Cloaciibacterium*	—	—	1.86	—	1.37	1.26
*Devosia*	—	2.29	—	—	—	—
*Exiguobacterium*	3.59	—	2.34	2.50	7.45	9.97
*Enterococcus*	0.64	—	—	—	1.11	2.17
*Ensifer*	—	3.61	—	—	—	—
*Flavobacterium*					0.96	1.33
*Lelliottia*	2.73	3.46	1.62	3.38	4.74	5.81
*Lysinibacillus*	—	—	—	—	2.25	1.69
*Mycobacterium*	42.68	0.17	13.74	9.75	0.44	—
*Methylobacterium*	—	13.98		2.55	1.30	—
*Mitochondria*	0.52	—	4.52	—	—	—
*Pseudomonas*	2.49	1.69	1.96	5.35	6.84	4.14
*Paracoccus*				2.18		
*Pantoea*	—	3.29	—	—	—	—
*Prevotella*			1.21			
*Rhodococcus*	13.44	13.86	17.60	10.06	7.66	12.23
*Ralstonia*	8.76	10.95	12.04	8.40	5.31	11.21
*Stenotrophomonas*	3.21	2.30	3.84	4.37	16.35	14.53
*Sphingomonas*	—	—	—	5.53	—	—
*Staphylococcus*	1.47					
*Trichococcus*	1.09	—	—	—	1.58	1.64
others	12.27	15.12	19.59	20.69	16.84	13.15

Venn diagram was used to identify the common and unique OTUs among different samples, OTU richness assessed from the 6 samples showed no obvious difference. The OTU abundance of *Dendrobium* stems from Huoshan was higher than *Dendrobium* stems from Yingshan on the whole. Samples from Huoshan and Yingshan shared 283 same OTUs, but harbored 237 and 135 unique OTUs respectively (Supplementary Fig. S3a). HM, HTie and HTong as samples from Huoshan shared 75 same OTUs, while harbored 134, 120 and 64 unique OTUs respectively (Supplementary Fig. S3b). YM, YTie and YTong as samples from Yingshan shared 73 same OTUs, while harbored 35, 88 and 130 unique OTUs respectively (Supplementary Fig. S3c). YM and HM shared 77 same OTUs, while harbored 58 and 190 unique OTUs respectively (Supplementary Fig. S3d). YTie and HTie shared 128 same OTUs, while harbored 108 and 171 unique OTUs respectively (Supplementary Fig. S3e). YTong and HTong shared 126 same OTUs, while harbored 159 and 105 unique OTUs respectively (Supplementary Fig. S3f).

**Figure 2:**
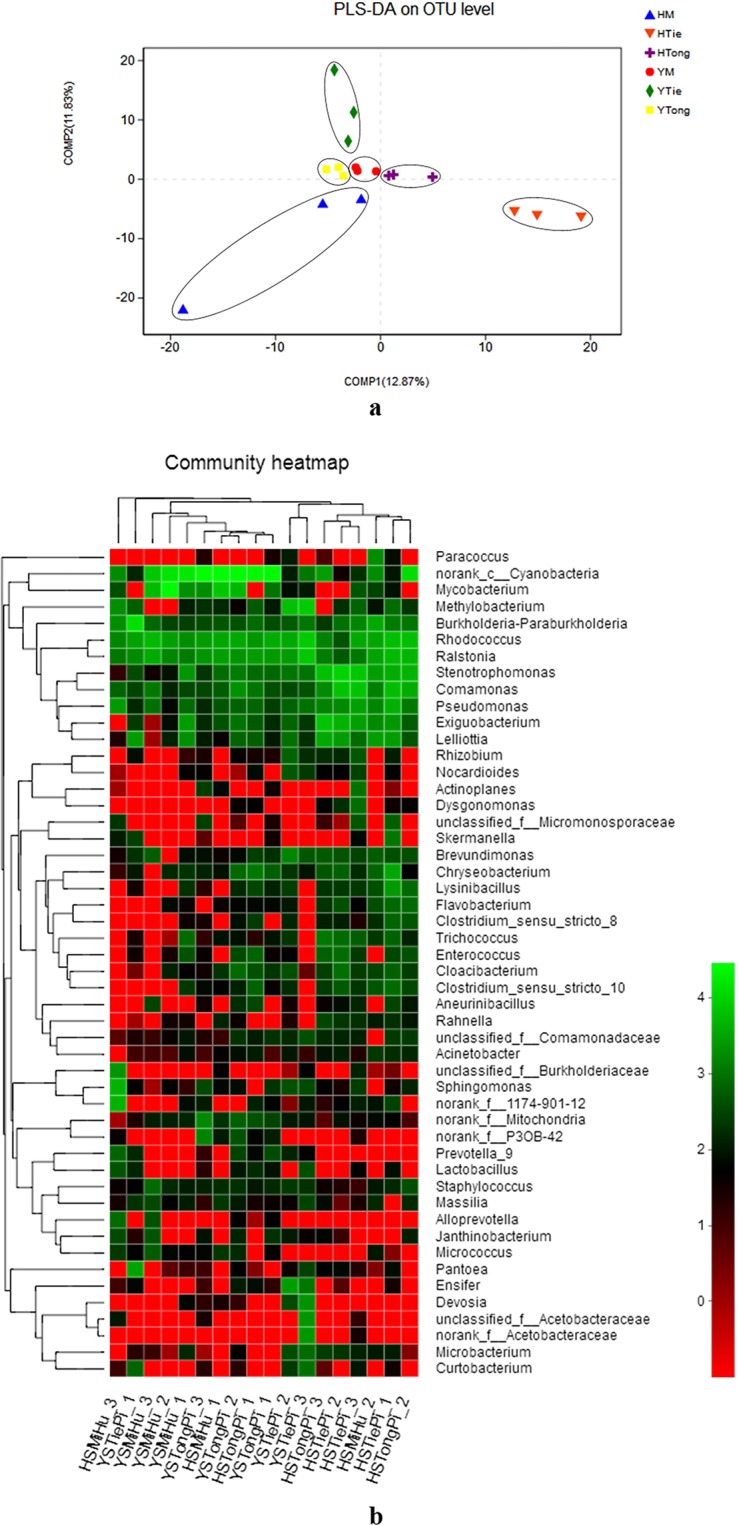
The differences among the samples in the grouping (**a**- *Partial Least Squares Discriminant Analysis* (PLS-DA) illustrates differences between bacterial communities in the 6 samples; **b**- Heatmap of the top 50 most abundant genera in bacterial communities detected in the 6 samples. Dendrograms for hierarchical cluster analysis grouping genera and sample locations are shown at the left and at the top, respectively).

In order to test the significance of inter-group differences among the samples in the grouping, *Partial Least Squares Discriminant Analysis* (PLS-DA) was used to conduct linear discrimination and classification modeling for grouping the samples. There were significant differences among different samples at the OTU level. The first principal coordinate separated the samples was based on the cultivars (COMP1). HM, HTie and HTong showed significant difference at level of COMP1, which showed an obvious difference about the bacteria community composition among cultivars of *Dendrobium*. It indicated that the cultivar was a factor to shape the community composition. While YM, YTie and YTong showed slight difference at the level of COMP1, which may require further investigation. On the level of COMP2, HM and YM, HTie and YTie showed significant difference, indicating that the origin also influences the shape of the endophytic community composition. While HTong and YTong showed significant difference at level of COMP1, which should be taken more attention (Fig. 2a).

A heatmap was drawn based on the distributions and abundances of the 6 samples on genus level (Fig. 2b). Hierarchical clustering analysis was used to detect the similarity of endophytic bacteria between samples. Based on the results, it was found that the samples from Houshan were clustered together, and other samples were located on the other branch except the HSTongPi_1, HSMiHu_1 and HSMiHu_3. So we deduced that the endophytic bacteria community was affected by their origins primarily.

#### Diversity of endophytic bacteria based on culture-dependent method

**Figure 3:**
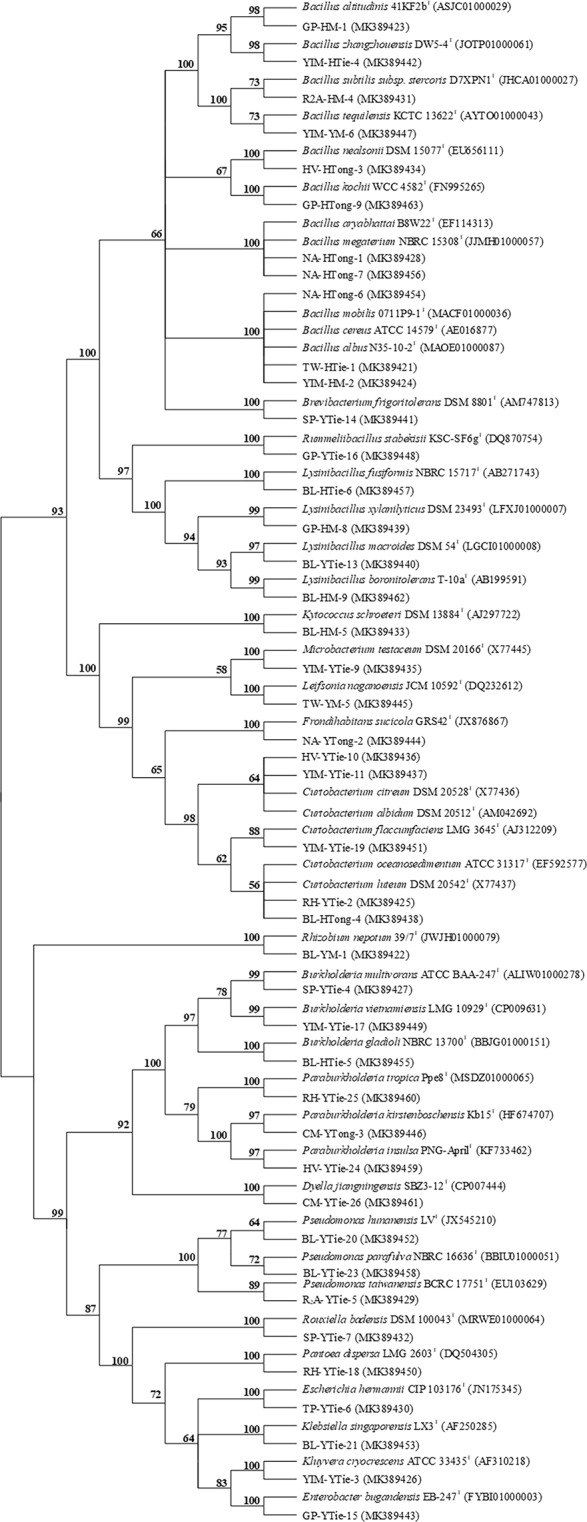
The phylogenetic tree of the 43 species identified in this study (GenBank accession numbers are given in parentheses. Strain name: medium type-sample type- strain number. For example: GP-HM-1 means that the bacterial strain No. 1 isolated from *D. huoshanense* collected from Huoshan using M-WA agar (GP) culture medium).

A total of 165 cultivable endophytic bacteria isolates were isolated from the sterilized *Dendrobium* stems, including 9 isolates from YM, 10 isolates from YTong, 106 isolates from YTie, 16 isolates from HM, 18 isolates from HTong and 6 isolates from HTie. All the isolated endophytic bacteria were identified by 16S rRNA gene sequence analysis, and then compared by the EzTaxon Database (https://www.ezbiocloud.net/). The endophytic bacteria were classified into 3 different phyla, 20 genera and 43 species. The 43 species were submitted to NCBI GenBank under Accession Number (MK389421- MK389463). The phylogenetic tree was shown in the Fig. 3.

**Figure 4:**
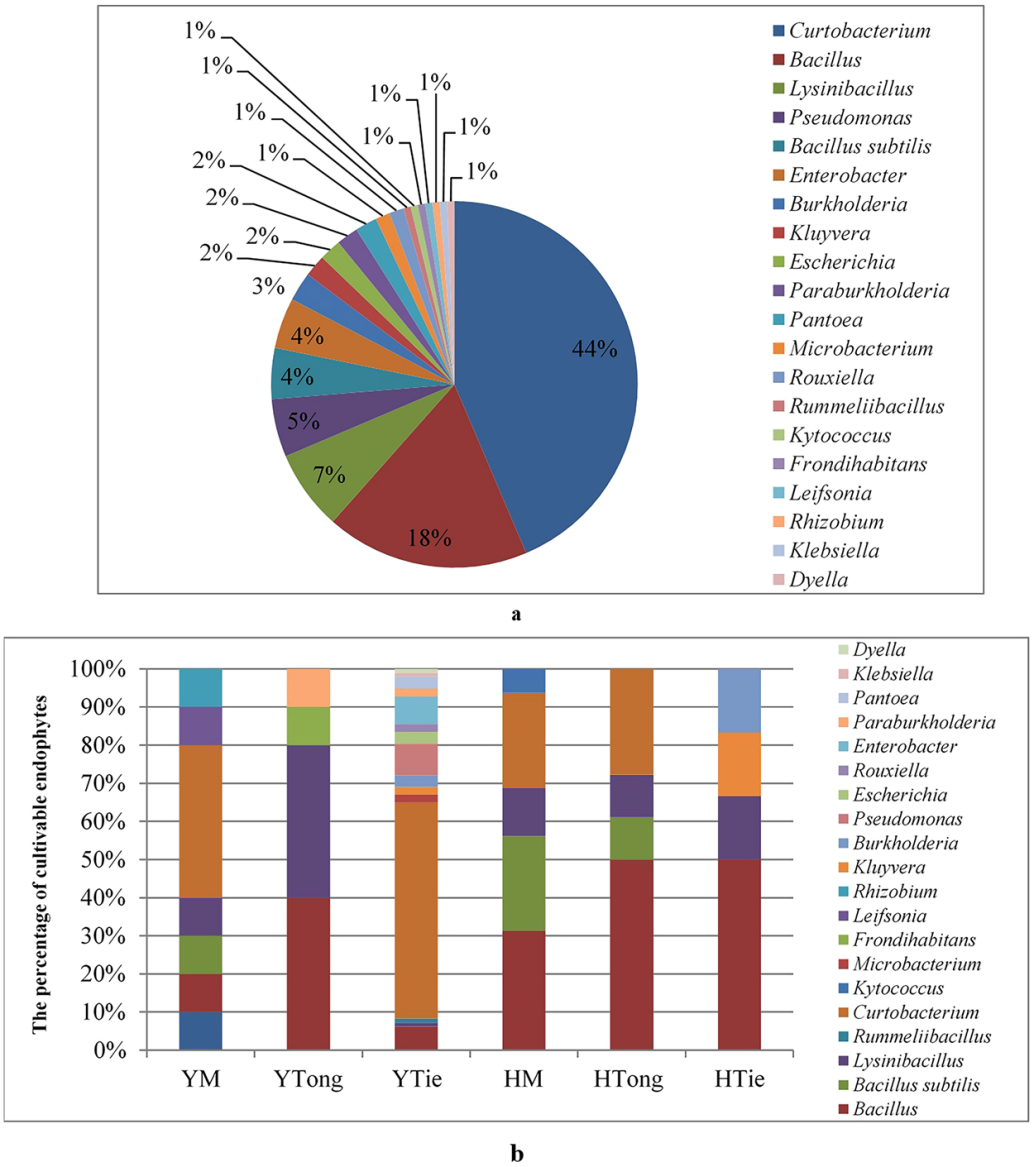
Diversity of endophytic bacteria composition of cultivable endophytes (**a** -Percent of cultivable endophytes on Genus level on the whole; **b** - Comparison of Genus level distributions for the 6 samples).

The 20 genera were dominated by *Curtobacterium* (44%) and *Bacillus* (18%). Except the predominant genera mentioned above, rare reported endophytic genera such as *Rouxiella*, *Rummeliibacillus*, *Kytococcus* were also appeared (Fig. 4a). Predominant endophytic bacteria for *Dendrobium* stems showed a slightly different result: the results showed that *Bacillus* (40.00%) and *Lysinibacillus* (40.00%) were predominant in YTong, while *Bacillus* (50.00%) was predominant in HTong. *Curtobacterium* (56.70%) was predominant in YTie, while *Bacillus* (50.00%) was predominant in HTie, which harbored very low-abundant endophytes compared to YTie. Interestingly, *Curtobacterium* (44.44%) was dominated in YM, while *Bacillus* (31.25%) was common in HM, this phenomenon is similar to YTie and HTie (Fig. 4b).

Obviously, the distribution of the cultivable endophytic bacterial community was influenced by the difference of origin and cultivar. The bacterial diversity of YTie was greater than any other *Dendrobium* stems. This result was consistent with the result of Illumina-based analysis.

#### Antimicrobial activity of endophytic bacteria against phytopathogen

The 43 species were tested for their antimicrobial activity against *Athelia rolfsii*, *Myrothecium roridum* and *Pectobacterium carotovorum* subsp. *actinidiae* using the Kirby-Bauer method. The supernatant and sediment were extracted separately used ethyl acetate and acetone. Among them, 14 strains showed antimicrobial activity against at least one pathogen in this study. These 14 strains belonged to 9 different genera, namely *Burkholderia*, *Bacillus*, *Paraburkholderia*, *Kluyvera*, *Rouxiella*, *Microbacterium*, *Brevibacterium*, *Pseudomonas* and *Dyella*. The genus *Bacillus* showed the highest antimicrobial activity against the selected pathogenic fungi and pathogenic bacteria. Out of the 14 strains, 1 strain was from HTie, 2 strains were from HTong, 1 strain was from YTong and 10 strains were from YTie (Table 2).

**Table 2:** Antimicrobial activity screening of endophytic bacteria against phytopathogen and their identification based on 16S rRNA gene sequencing analysis.

Strain	Number	GenBank accession number	Closest species in 16S rRNA gene sequences database	samilarity%	Active part	Pathogenic bacteria	Pathogenic fungi	
						*Pectobacterium carotovorum* subsp*. actinidiae*	*Athelia rolfsii*	*Myrothecium roridum*
228	BL-HTie-5	MK389455	*Burkholderia gladioli*	98.93	supernatant	−	+++	+++
					sediment	−	+++	+++
214	NA-HTong-6	MK389454	*Bacillus albus*	98.88	supernatant	−	+	++
					sediment	−	++	++
207	NA-HTong-7	MK389456	*Bacillus megaterium*	99.12	supernatant	++	+++	+++
					sediment	−	+++	+++
65	CM-YTong-3	MK389446	*Paraburkholderia kirstenboschensis*	98.87	supernatant	−	+	+
					sediment	−	−	−
166	YIM-YTie-3	MK389426	*Kluyvera cryocrescens*	98.43	supernatant	−	−	+
					sediment	−	−	−
70	SP-YTie-7	MK389432	*Rouxiella badensis*	99.8	supernatant	−	++	++
					sediment	−	+	+
97	YIM-YTie-9	MK389435	*Microbacterium testaceum*	98.78	supernatant	−	++	++
					sediment	−	−	+
13	SP-YTie-14	MK389441	*Brevibacterium frigoritolerans*	99.93	supernatant	−	+	+
					sediment	−	−	−
124	YIM-YTie-17	MK389449	*Burkholderia vietnamiensis*	97.48	supernatant	−	−	−
					sediment	−	−	++
107	BL-YTie-20	MK389452	*Pseudomonas hunanensis*	99.79	supernatant	−	−	+
					sediment	−	−	−
236	BL-YTie-23	MK389458	*Pseudomonas parafulva*	99.29	supernatant	−	−	+
					sediment	−	−	−
194	HV-YTie-24	MK389459	*Paraburkholderia insulsa*	99.02	supernatant	−	+	−
					sediment	−	+	−
202	RH-YTie-25	MK389460	*Paraburkholderia tropica*	99.04	supernatant	−	−	−
					sediment	−	+	+
135	CM-YTie-26	MK389461	*Dyella jiangningensis*	99.64	supernatant	−	++	+
					sediment	−	++	−

− indicates no activity; +, indicates positive activity, diameter of zones of inhibition less than 10 mm; ++, indicates medium activity, diameter of zones of inhibition between 10 and 20 mm; +++, indicates strong activity, diameter of zones of inhibition greater than 20 mm.

In the assay of ethyl acetate extracts of the supernatant, only NA-HTong-7 showed antimicrobial activity against all three phytopathogens, and its inhibitory activity was better than the 70% Mancozeb (750 fold dilution) (Supplementary Fig. S4). While other strains showed inhibition activity against at least one of the pathogenic fungi, except YIM-YTie-17 and RH-YTie-25 which showed no activity to any of the phytopathogens. Especially, the extracts of NA-HTong-7 and BL-HTie-5 showed the highest antifungal activity against *Athelia rolfsii* and *Myrothecium roridum*, with the diameter of zones of inhibition greater than 20 mm.

In the assay of acetone extracts of the sediment, all the isolates showed no antimicrobial activity against the pathogenic bacteria. Interestingly, the extracts of NA-HTong-7 and BL-HTie-5 also showed the highest antifungal activity against *Athelia rolfsii* and *Myrothecium roridum*. The extracts of CM-YTong-3, YIM-YTie-3, SP-YTie-14, BL-YTie-20 and BL-YTie-23 showed no antimicrobial activity against any of the three phytopathogens, while the extracts of NA-HTong-6, SP-YTie-7, YIM-YTie-9, YIM-YTie-17, HV-YTie-24, RH-YTie-25 and CM-YTie-26 showed inhibition activity against at least one of the pathogenic fungi.

## Discussion

In this study, the great diversity of endophytic bacteria with a considerable number of antimicrobial strains in the 6 *Dendrobium* stems was demonstrated, and the results showed that the distribution of the endophytic bacterial community was influenced by the difference of origins and cultivars. Meanwhile, the culture-dependent and culture-independent methods of the endophytic bacteria diversity of *Dendrobium* stems were evaluated. The results will provide new insights into the endophytic bacteria from *Dendrobium*.

**Figure 5:**
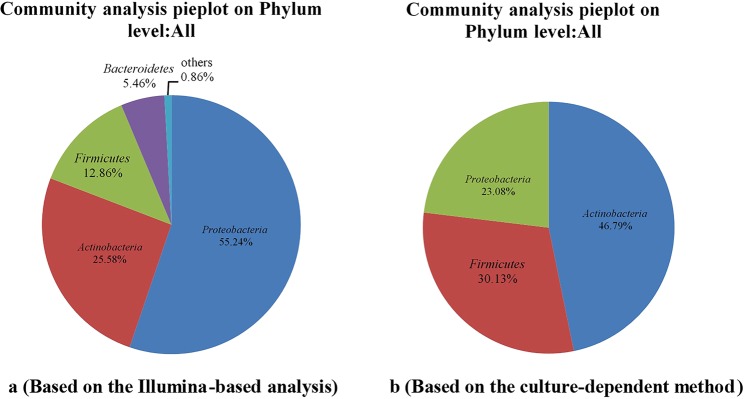
Composition and relative abundance of endophytic bacterial phyla in different samples (**a** -Percent of the culture-independent endophytic bacteria on phylum level on the whole; **b** - Percent of the cultivable endophytic bacteria on phylum level on the whole).

According to the results, differences were found between the two methods, especially on the genus level. It is easy to detect redundant more genera using Illumina-based analysis method and thus it is a more reliable method for investigating the endogenous microorganisms communities of plants. Twenty-two different phyla were detected based on the Illumina-based analysis. *Proteobacteria* (55.24%), *Actinobacteria* (25.58%), *Firmicutes* (12.86%) and *Bacteroidetes* (5.46%) were the four most dominant phyla, accounting for 99.14% of the reads (Fig. 5a). While the cultivable endophytic bacteria were classified into 3 different phyla, including *Actinobacteria* (46.79%), *Firmicutes* (30.13%), and *Proteobacteria* (23.08%) (Fig. 5b). Compared with the results of Illumina-based analysis, *Bacteroidetes* was not isolated by the culture-dependent method. It’s worth noting that *Proteobacteria* was predominant in the 6 *Dendrobium* stems for the Illumina-based analysis, while occupied a smaller proportion based on the culture-dependent method.

On the genus level, there were 23 genera identified through culture-dependent methods, while 373 genera were sequenced from the 6 *Dendrobium* stems based on Illumina-based analysis, with 15 of the genera having an average abundance of over 1% (Supplementary Fig. S5). The significantly higher proportion of *Curtobacterium* (44%) and *Bacillis* (18%) were observed in the 6 *Dendrobium* stems by the culture-dependent method. However, *Rhodococcus* (11.84%) was occupied high abundance as revealed through the Illumina-based analysis. By the culture-dependent method, the isolates from *Dendrobium* stems included *Bacillus*, *Enterobacter*, *Klebsiella*, *Pantoea*, *Pseudomonas*, *Curtobacterium*, *Burkholderia*, *Microbacterium* and *Lysinibacillus*, which have been reported in previous studies^22–25^. While some bacterial genera that have not been reported from *Dendrobium* were detected by Illumina-based analysis, including *Ralstonia*, *Comamonas* and *Lelliottia*, which indicated that there is still a lot of research space for endophytes of *Dendrobium*^26,27^. It is evident that the culture-dependent method tended to produce biased results for microbes. The culturable bacteria were probably the largest, most active bacteria in *Dendrobium* samples^28–30^. A combination of culture-dependent and culture-independent methods might provide a powerful strategy to investigate microbes, which will help us identify and obtain some novel or difficult-to-cultivate endophytes.

In total, 165 cultivable endophytic bacteria were isolated and assigned into 20 genera dominanted by *Curtobacterium* (44%) and *Bacillis* (18%). Eleven different types of media were used to isolate endophytic bacteria from sterilized *Dendrobium* stems, and all media were supplemented with 1% plant extracts of *Dendrobium* stems to offer natural growth conditions for the potential endophytes in plant tissues to obtain more culturable single colonies. In fact, the genera preference of cultivable endophytic bacteria was influenced by the isolation media. According to the results, differences were found among the 11 different media (Supplementary Fig. S6). Among the media used in the experiment, YIM38 was proven to be most favorable to the growth of cultivable endophytic bacteria, with 31 strains were isolated from it. On the other hand, the endophytic strains isolated from BL medium showed higher diversity compared to other 10 media, which demonstrated that BL medium possessed more extensive adaptability for bacterial growth. It’s worth noting that *Curtobacterium* was the only genus isolated from all the media. The influence of different media components on endophytes diversity was showed in this study, especially the factors such as Carbon source and Nitrogen source. It suggested that environmental factors such as oxygen content should be taken more attention in investigations of plant endophytes.

Combining with culture-dependent and culture-independent methods, the endophytic bacterial community in *Dendrobium* stems was different in the 6 samples and the distribution of the endophytic bacterial community was influenced by the difference of origins and cultivars, but mostly the origins. This result agreed with research that endophytic bacterium strains are obtained from different *Dendrobium* collected from different locations showed specificity distribution characteristics, and the specific endophytic bacteria promote the formation of geo-authentic crude drug^31,32^.

Endophytes associated with medicinal plants may produce the same metabolites *in vitro* and within host plant tissue^33,34^, which also are the rich sources of secondary metabolites with antimicrobial activity. In this study, the antimicrobial activity of a diverse collection of endophytes isolated from *Dendrobium* stems, consisting of 20 genera and 43 species was analyzed. *Athelia rolfsii*, *Myrothecium roridum* as pathogenic fungi of *Dendrobium*, and *Pectobacterium carotovorum* subsp. *actinidiae* as pathogenic bacteria of *Dendrobium*, were used to determine the antimicrobial activities. In these assays, a significant fraction of the endophytic bacteria (32.6%) displayed antagonistic effects. Among the 43 species, 14 strains harbored at least one positive antimicrobial activity in this study. These 14 strains belonged to 9 different genera, but only genus *Bacillus* showed the highest antimicrobial activity against all three indicator pathogens. Previous studies have demonstrated that *Bacillus* strains associated with other medicinal plants exhibited antibacterial activity not only against phytopathogens such as *Fusarium moniliforme*, *Fusarium oxysporumf*.sp*.vasinfectum*, *Verticillium dahlia* and *Burkholderia solanacearum*^35–38^, but also the 3 pathogens used in this study^39–42^. The genus *Bacillus* is well known for the natural production of secondary metabolites with antibacterial and antifungal activities and has a strong potential to control plant diseases^43–45^. Our study further illustrates its potential role as a biological agent for controlling phytopathogens.

At present, the research on endophytes of *Dendrobium* mainly focuses on the isolation of endophytes of a single *Dendrobium* sample. However, to realize the potential of beneficial microbes for medicinal herb applications, research should be carried out to study variations in the effective components of the same cultivar from different origins or the same origin from different cultivars. In our study, we have combined the culture-dependent and culture-independent methods to explore the difference of endophytic bacteria of the 6 *Dendrobium* samples. The result demonstrated that there was a great diversity of endophytic bacteria in the 6 *Dendrobium* stems, with a considerable number of antimicrobial strains, and showed that the distribution of the endophytic bacterial community was influenced by the difference of origins and cultivars, especially the origins. However, we have not studied the relationship between the medicinal components of *Dendrobium* and the endophytes, which may be the further research plan in our laboratory.

## Materials and Methods

### Plant sampling and surface sterilization

*Dendrobium* samples were collected from Yingshan, Hubei Province, China and Huoshan, Anhui Province, China. The plant samples included 6 cultivars originated from the two different places (Table 3). Stems were collected from 6 cultivars of *Dendrobium* samples randomly. The plant samples were randomly divided into three groups placed in three different plastic bags. All 18 plant samples were transported to the laboratory at 4 °C. *Dendrobium* samples were labeled according to the difference of origins and cultivars as YM, YTong, YTie, HM, HTong, HTie.

**Table 3:** *Dendrobium* samples codes and their origin.

Samples	Species	Tissue	Latitude	Longitude	Location
YM	*D. huoshanense*	stem	115°31′~116°04′	30°00′~31°08′	Yingshan, Hubei Province, China
YTong	*D. moniliforme*	stem			
YTie	*D. officinale*	stem			
HM	*D. huoshanense*	stem	115°52′~116°32′	31°03′~31°33′	Huoshan, Anhui Province, China
HTong	*D. moniliforme*	stem			
Htie	*D. officinale*	stem			

In order to avoid the influence of microorganisms on the surface of *Dendrobium*, the stem samples of *Dendrobium* were surface-sterilized as follows: *Dendrobium* stems were washed by tap water and underwent ultrasonic treatment (40 kHz) for 15 min. Then surface sterilized with 1% (v/v) Tween 20 for 1 min; 2.5% (w/v) NaS_2_O_3_ for 10 min; 75% ethanol for 5 min. Next, stems were rinsed with distilled water three times. To confirm the sterilization process was successful, 200 μL of the final water rinse was plated on the eleven different media along with 1% plant extracts of *Dendrobium* stems (Supplementary Table S2), which consistently yielded no bacterial colonies incubated at 28 °C for two weeks.

### Illumina-based analysis of endophytic bacterial

#### DNA extraction and PCR amplification

Microbial DNA was extracted from the samples using the E.Z.N.A.® Bacterial DNA Kit (Omega Bio-tek, Norcross, GA, U.S.) according to the manufacturer’s protocols. The final DNA concentration and purification were determined by NanoDrop 2000 UV-vis spectrophotometer (Thermo Scientific, Wilmington, USA), and DNA quality was checked by 1% agarose gel electrophoresis.

The V5–V7 regions of the bacteria 16S rRNA gene of each sample were amplified for twice. The PCR reactions were first conducted with primers 799 f (5′-AACMGGATTAGATACCCKG-3′) and 1392r (5′-ACGGGCGGTGTGTRC-3′) by thermocycler PCR system (GeneAmp 9700, ABI, USA). Subsequently, the initial PCR products were used as templates for the second amplification with primers 799 f (5′-AACMGGATTAGATACCCKG-3′) and 1193r (5′-ACGTCATCCCCACC TTCC-3′). The PCR reactions were conducted using the standard procedure^46^, with slight modifications. PCR reactions were performed in 20 μL mixture containing 4 μL of 5 × FastPfu Buffer, 2 μL of 2.5 mM dNTPs, 0.8 μL of each primer (5 μM), 0.2 μL of BSA, 0.4 μL of FastPfu Polymerase and 10 ng of template DNA, and then, adding up to 20 μL with ddH_2_O.

#### Illumina MiSeq sequencing

The resulted PCR products were extracted from a 2% agarose gel and further purified using the AxyPrep DNA Gel Extraction Kit (Axygen Biosciences, Union City, CA, USA) and quantified using QuantiFluor™-ST (Promega, USA) according to the manufacturer’s protocol. Purified amplicons were pooled in equimolar and paired-end sequenced (2 × 250 bp) on an Illumina MiSeq platform (Illumina, San Diego,USA) according to the standard protocols by Majorbio Bio-Pharm Technology Co. Ltd. (Shanghai, China). Sequencing was by using Illumina’s Miseq PE250 platform.

#### Processing of sequencing data

Raw FASTQ files were quality-filtered by Trimmomatic and merged by FLASH by the standard procedure^47^. Operational taxonomic units (OTUs) were clustered with 97% similarity cutoff using UPARSE (version 7.1 http://drive5.com/uparse/) with a novel ‘greedy’ algorithm that performs chimera filtering and OTU clustering simultaneously. The taxonomy of each 16S rRNA gene sequence was analyzed by RDP Classifier algorithm (http://rdp.cme.msu.edu/) against the Silva (SSU123) 16SrRNA database using confidence threshold of 70%.

#### Data analysis

Rarefaction curves were plotted for each sample to determine the abundance of communities and sequencing data of each sample. Alpha-diversity analyses, including community richness parameters (Chao, Ace, Sobs), community diversity parameters (Shannon, Simpson) and Community coverage index (Coverage), were calculated using the mothur software. Bacterial taxonomic distributions of sample communities were visualized using the R package software. Venn diagram was implemented using the R package to show unique and shared OTUs. Partial Least Squares Discriminant Analysis (PLS-DA) based on OTU compositions were determined. Student’s t-test was used to detect whether there was a significant difference in The Observed Richness Value (Sobs) between the samples.

### Isolation and identification of endophytic bacteria

#### Isolation of Endophytic bacteria from *Dendrobium* stems

The sterilized *Dendrobium* stems were cut by sterile surgical scissors into 0.5~1.0 cm segments, and placed onto different types of media plates to obtain more culturable single colonies. All media were supplemented with 1% plant extracts of *Dendrobium* stems to offer natural growth conditions for the potential endophytes in plant tissues. The plates were incubated at 28 °C until emergence of bacterial colonies from inside the samples.

Single-colony isolation was repeated at least three times for purification of each of the endophytic bacteria isolates by use of the YIM38 agar medium. Finally, the selected endophytic bacteria isolates were stored in 50% (v/v) glycerol (Bacterial fluid: 50% glycerol = 1:1, v/v) at −80 °C for further study.

#### Identification of Endophytic bacteria

The endophytic microbial DNA was extracted following the standard procedure^46^, with slight modifications. Bacterial 16S rRNA genes were amplified using the genomic DNA as template and bacterial universal primers of 27 f and 1492r by thermocycler PCR system (Applied Biosystems, Singapore). The PCR reaction were performed in 50 μL mixture containing 25 μL of 1 × EasyTap PCR SuperMix (TransGen Biotech), 1.5 μL of universal primers 27 f, 1.5 μL of universal primers 1492r, 20 μL of sterilized water, 2 μL of genomic DNA. The PCR amplification procedure was shown as follow (Supplementary Table S3). The PCR products were checked for the expected size on 1.2% agarose gel and were sequenced at Sangon Lab (Shanghai, China) with the two primers.

The EzTaxon database (https://www.ezbiocloud.net/) was used to compare the 16S rRNA gene sequences of the endophytic bacterial strains, and the sequences were submitted to NCBI Genbank. The phylogenetic tree was constructed using the Neighbour-Joining method through the Mega7 software.

### Screening for antimicrobial activity of endophytic bacteria against phytopathogen

#### Collection of the solvent extract of endophytic bacteria

The single colony of selected endophytic bacteria were picked and inoculated into 150 mL of the YIM38 liquid medium in 500 mL Erlenmeyer flasks followed by incubation with continuous shaking at 28 °C for 2 days at 200 rpm, while the single colony of selected endophytic actinomycetes were shaking for 7 days under the same conditions. The supernatant and sediment were collected separately after the culture broth was centrifuged at 9500 rpm for 20 min at 4 °C. The supernatant was mixed with an equal volume of ethyl acetate, then the ethyl acetate layer was collected by means of a separating funnel and dried using a rotary evaporator (RE-52AA; YARONG, China) at 38 °C. The sediment mixed with 15 mL of acetone for 12 h was filtered through a 0.22 μm nylon membrane filter and dried by using a rotary evaporator (RE-52AA; YARONG, China) at 38 °C. The extracts were then redissolved in 1 mL of methanol and stored at −20 °C for antimicrobial activity screening.

#### Screening for antimicrobial activity

*Athelia rolfsii*, and *Myrothecium roridum* as pathogenic fungi of *Dendrobium*, and *Pectobacterium carotovorum* subsp. *actinidiae* as pathogenic bacteria of *Dendrobium*, were used in the present study to determine the antimicrobial activities of the solvent extract of endophytic bacteria (Supplementary Table S4). These phytopathogens were obtained from the Sanming Academy Of Agricultural Sciences, Fujian Province, China.

The Kirby-Bauer test was used to screen for antimicrobial activity. Respectively, 10 mL cultures of the pathogenic fungi grown 2 days in the Potato Dextrose (PD) liquid medium at 28 °C was added to the 100 ml of the PD agar medium, while 10 mL cultures of the pathogenic bacteria grown 12 h in the Luria – Bertani (LB) liquid medium at 28 °C was added to the 100 ml of the LB agar medium, mixed gently, and then poured slowly on the petri dish used as the test plate. Sterilized paper discs (diameter, 6 mm) were saturated with the solvent extract (20 μL) and placed onto the test plates inoculated with the test organism. An equivalent volume of methanol instead of the endophytic bacteria was used as a negative control, and an equivalent volume of 70% Mancozeb (750 fold dilution) was used as a positive control. Plates were then incubated 16 h under aerobic conditions at 28 °C. The effect of antimicrobial activity was determined by measuring the diameter of the inhibition zones (using electronic digital caliper, 0–150 mm).

## Supplementary information

Supplementary Information

## Data Availability

All data underlying this publication are available in the manuscript.
