# Supplementary material for: Diversity of culture-independent bacteria and antimicrobial activity of culturable endophytic bacteria isolated from different Dendrobium stems
Source: Sci Rep. 2019 Jul 17;9:10389. doi: 10.1038/s41598-019-46863-9 (PMC6637234; doi:10.1038/s41598-019-46863-9)
Supplement: Supplementary file 1 — Supplementary Information [file 41598_2019_46863_MOESM1_ESM.docx]

**Diversity of culture-independent bacteria and antimicrobial activity of culturable endophytic bacteria isolated from different *Dendrobium* stems**

Shan-shan Wang ^a^, Jia-meng Liu^a,^*, Jing Sun, Yu-feng Sun, Jia-ni Liu, Ning Jia, Bei Fan*, Xiao-feng Dai*

**a: These authors have contributed equally to this work.**

***Correspondence:** **Jia-meng Liu** **ljiam88@sina.com**

**Bei Fan** [**caasfanbei@163.com**](mailto:caasfanbei@163.com)

**Xiao-feng Dai** [**CAASXiaofengDai@163.com**](mailto:CAASXiaofengDai@163.com)

Key Laboratory of Agro-products Quality and Safety Control in Storage and Transport Process, Ministry of Agriculture, Beijing, China / Chinese Academy of Agricultural Science, Institute of Food Science and Technology CAAS, Beijing, China

**Supplementary Table S1. Number of OUTs and Alpha diversity of endophytic bacteria in *Dendrobium* samples**

| Sample | Reads | OTUs | Alpha diversity indices | | | | | |
| --- | --- | --- | --- | --- | --- | --- | --- | --- |
|  |  |  | Sobs | Shannon | Simpson | Ace | Chao | Coverage |
| YSMiHu_1 | 33630 | 92 | 92 | 2.45 | 0.23 | 94.50 | 95.00 | 99.99% |
| YSMiHu_2 | 46464 | 70 | 70 | 1.52 | 0.36 | 126.53 | 125.00 | 99.98% |
| YSMiHu_3 | 31650 | 47 | 47 | 2.54 | 0.13 | 130.64 | 59.00 | 99.97% |
| YSTongPi_1 | 40239 | 157 | 157 | 2.80 | 0.16 | 160.33 | 160.00 | 99.99% |
| YSTongPi_2 | 50682 | 159 | 159 | 2.94 | 0.14 | 165.70 | 173.00 | 99.98% |
| YSTongPi_3 | 43689 | 159 | 159 | 2.40 | 0.24 | 162.32 | 164.00 | 99.99% |
| YSTiePi_1 | 30188 | 104 | 104 | 2.39 | 0.22 | 125.78 | 115.25 | 99.97% |
| YSTiePi_2 | 29790 | 125 | 125 | 3.48 | 0.07 | 131.70 | 129.20 | 99.98% |
| YSTiePi_3 | 48034 | 136 | 136 | 3.25 | 0.09 | 149.71 | 158.00 | 99.98% |
| HSMiHu_1 | 46869 | 85 | 85 | 1.99 | 0.32 | 90.75 | 88.33 | 99.99% |
| HSMiHu_2 | 30754 | 79 | 79 | 3.27 | 0.06 | 83.51 | 82.33 | 99.98% |
| HSMiHu_3 | 34306 | 183 | 183 | 3.88 | 0.04 | 186.32 | 190.00 | 99.98% |
| HSTongPi_1 | 41633 | 102 | 102 | 2.70 | 0.20 | 109.49 | 105.75 | 99.99% |
| HSTongPi_2 | 42366 | 78 | 78 | 2.95 | 0.09 | 81.52 | 81.33 | 99.99% |
| HSTongPi_3 | 33082 | 160 | 160 | 3.27 | 0.08 | 161.53 | 175.00 | 99.98% |
| HSTiePi_1 | 54747 | 148 | 148 | 3.66 | 0.05 | 159.75 | 155.50 | 99.98% |
| HSTiePi_2 | 36693 | 170 | 170 | 3.14 | 0.09 | 173.12 | 179.00 | 99.98% |
| HSTiePi_3 | 38606 | 170 | 170 | 3.44 | 0.07 | 172.77 | 175.00 | 99.98% |

**Supplementary Table S2. Nutrient composition of the 11 different media used in this study**

| **Medium name** | **Nutrient Composition** |
| --- | --- |
| Humic acid agar (HV) | CaCO_3_ 0.02 g, Humic acid 1.0 g, KCl 1.7 g, FeSO_4_•7H_2_O 0.01 g, MgSO_4_•7H_2_O 0.05 g, Na_2_HPO_4_ 0.5 g, Agar 20.0 g, dd H_2_O 1 L |
| 10% YIM38 agar (YIM38) | Glucose 0.4 g, Yeast extract 0.4 g, Malt extract 0.5 g, B-Vitamins 0.1 mL / L, Trace salt 0.1 mL / L, Agar 20.0 g, dd H_2_O 1 L |
| Tap water yeast glucose starch agar (TW) | Yeast extract 0.25 g, K_2_HPO_4_ 0.5 g, Agar 20.0 g, Tap water 1 L |
| Glucose starch agar (BL) | Glucose 5.0 g, Yeast extract 5.0 g, Soluble starch 5.0 g, Casamino acids hydrolysate 2.0 g, CaCO_3_ 5.0 g, NaCl 5.0 g, Agar 20.0 g, dd H_2_O 1 L |
| Sodium propionate agar (SP) | CaCl_2_•2H_2_O 0.02g, Sodium propionate 1.0 g, L-Asparagine 0.2 g, K_2_HPO_4_ 0.6 g, KH_2_PO_4_ 0.9 g, MgSO_4_•7H_2_O 0.1 g, Agar 20.0 g, dd H_2_O 1 L |
| Trehalose – proline agar (TP) | CaCl_2_ 2.0 g, (NH_4_)_2_SO_4_ 1.0 g, Trehalose 5.0 g, Proline1.0 g, NaCl 1.0 g, K_2_HPO_4_ 1.0 g, MgSO_4_•7H_2_O 1.0 g, B-Vitamins 1 mL, Agar 20.0 g, dd H_2_O 1 L |
| Cellulose agar (CM) | CaCO_3_ 0.02 g, KNO_3_ 0.2 g, Cellulose 10.0 g, Casein 0.3 g, K_2_HPO_4_ 0.2 g, FeSO_4_ 0.01 g, MgSO_4_•7H_2_O 0.05 g, Agar 20.0 g, dd H_2_O 1 L |
| M-WA agar (GP) | Glycerol 10.0 g, Yeast extract 0.5 g, KNO_3_ 0.5 g, Proline1.0 g, L-Asparagine 1.0 g, Sodium pyruvate 1.25 g, Betaine 1.25 g, Agar 20.0 g, dd H_2_O 1 L |
| 10% Nutrient agar (NA) | Beef extract 0.5 g, Peptone 1 g, Sodium pyruvate 1.25 g, Betaine 1.25 g, NaCl 10 g, Agar 20.0 g, dd H_2_O 1 L |
| Raffinose–histidine agar (RH) | Raffinose 1.0 g, CaCO_3_ 0.02 g, Histidine 0.1 g, Na_2_HPO_4_ 0.5 g, KCl 1.7 g, MgSO_4_•7H_2_O 0.05 g, FeSO_4_•7H_2_O 0.1 g, B-Viramins 1 mL, Agar 20.0 g, dd H_2_O 1 L |
| R_2_A agar (R_2_A) | Glucose 0.5 g, Yeast extract 0.5 g, Peptone 0.5 g, Casein peptone 0.5g, Sodium pyruvate 0.3g, MgSO_4_•7H_2_O 0.024 g, K_2_HPO_4_ 0.3 g, Soluble starch 0.5g, Agar 20.0 g, dd H_2_O 1 L |
| PH:7.0 Sterilizing at 121°C for 15 min | |

**Supplementary Table S3. PCR amplification procedure**

| **Reaction procedure** | **Temperature/°C** | **Time/min** |  |
| --- | --- | --- | --- |
| Pre-degeneration | 95 | 5 |  |
| Denaturation | 94 | 1 | 35 cycles |
| Anneal | 55 | 1 |  |
| Extend | 72 | 2 |  |
| Final extend | 72 | 10 |  |

**Supplementary Table S4. Phytopathogen used in this study**

| **Scientific Name** | **Type strain** | **Gram reaction** | **Disease** |
| --- | --- | --- | --- |
| *Athelia rolfsii* | HQ420816.1 | — | *Southern blight* |
| *Myrothecium roridum* | ATCC 60379 | Gram- positive | *Tar spot disease* |
| *Pectobacterium carotovorum* subsp. *actinidiae* | KKH3^T^ (=KCTC  23131^T^=LMG 26003^T^) | Gram-negative | *Soft rot* |


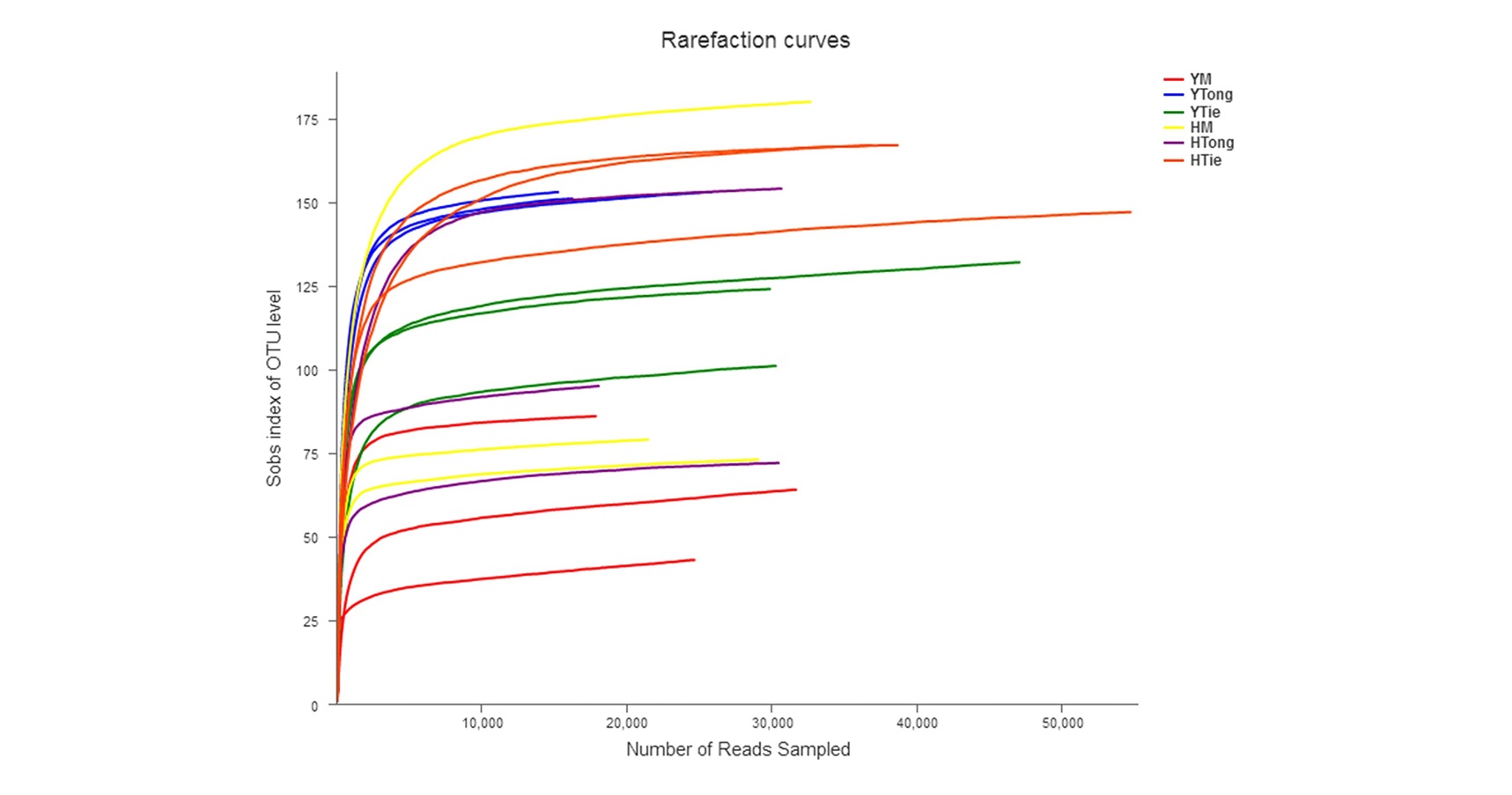


**Supplementary Fig.S1-Rarefaction curves of the OTU number at 97% similarity for different samples.** **The rarefaction curves showed that the sequencing work was relatively comprehensive in covering the bacterial diversity, as the curves tended to approach saturation, indicating that the selected sequence data adequately reflected the bacterial abundance of these samples.**
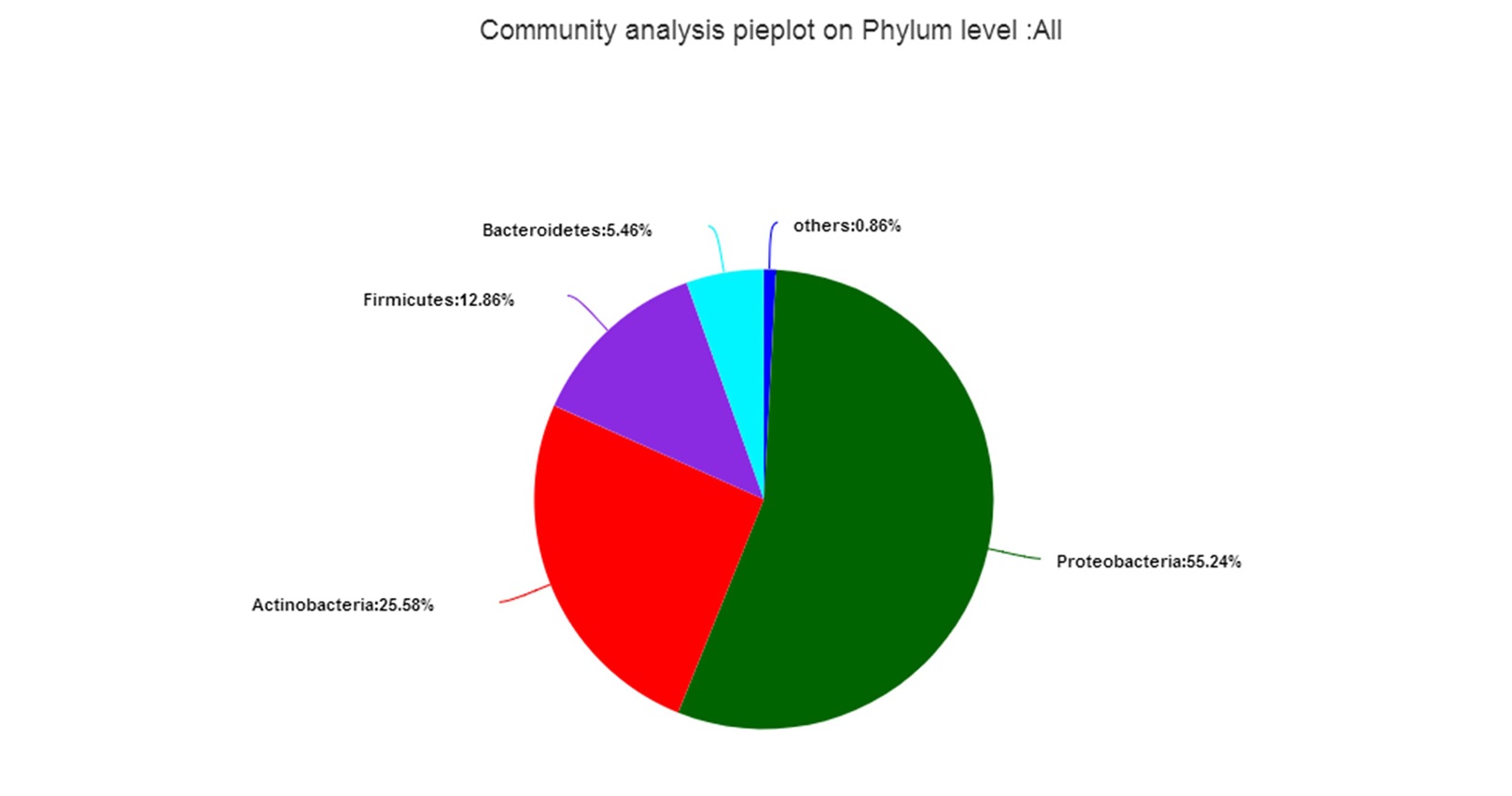


**Supplementary Fig. S2-Composition and relative abundance of endophytic bacterial in different samples on phylum level. Phyla making up less than 1% of total composition in the samples were classified as “other”.**

**
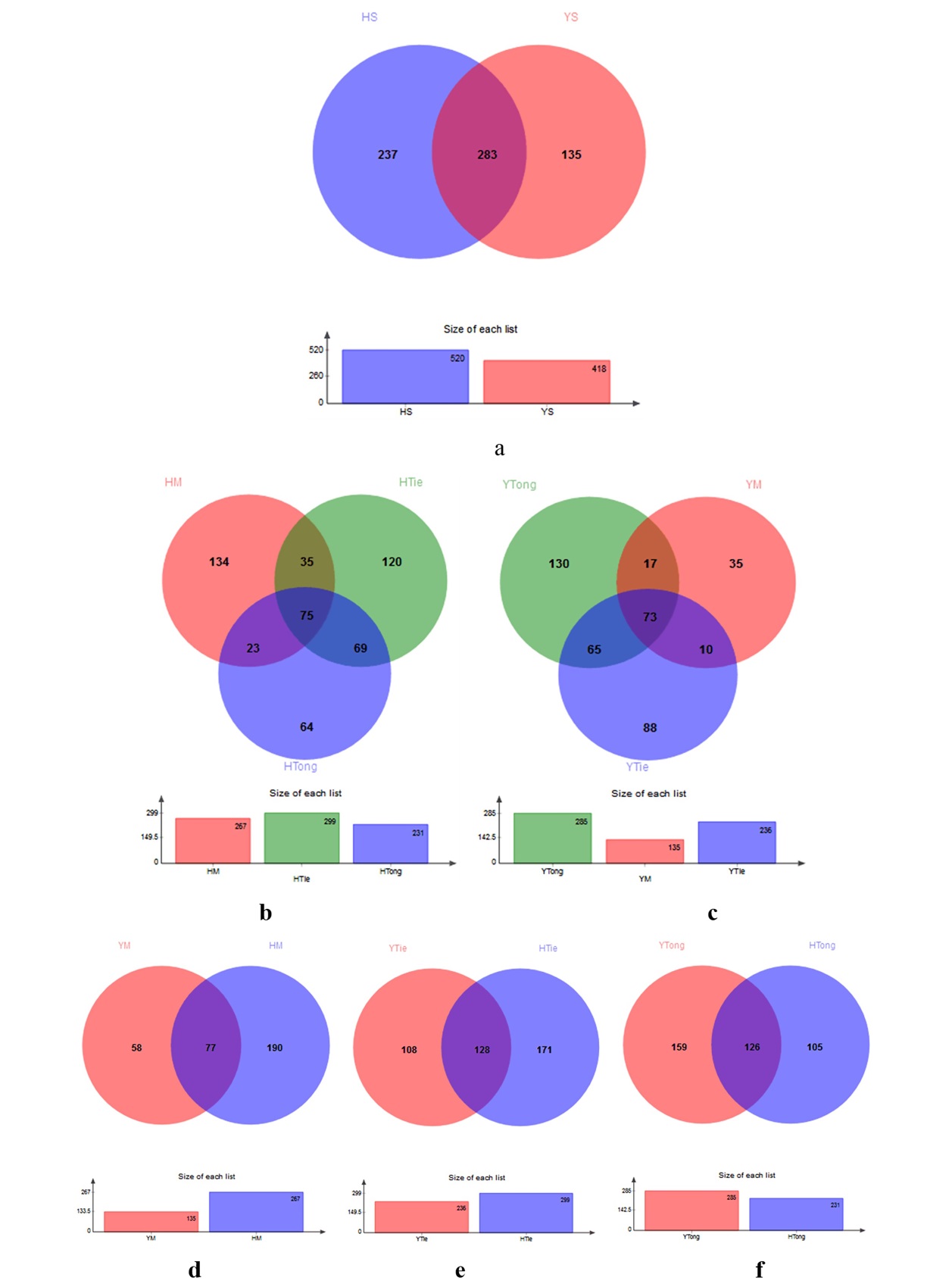
Supplementary Fig.S3-Venn diagrams showing the number of OTUs shared and unique among different samples (Fig.S3a - The common and unique OTUs between the endophytic bacteria of *Dendrobium* stems from Huoshan and Yingshan; Fig.S3b - The common and unique OTUs among the endophytic bacteria of the three samples from Huoshan; Fig.S3c - The common and unique OTUs among the endophytic bacteria of the three samples from Yingshan; Fig.S3d - The common and unique OTUs between the endophytic bacteria of the *D. huoshanense* from Huoshan and Yingshan; Fig.S3e - The common and unique OTUs** **between the endophytic bacteria of the *D. officinale* from Huoshan and Yingshan; Fig.S3f - The common and unique OTUs between the endophytic bacteria of the *D. moniliforme* from Huoshan and Yingshan.)**

**
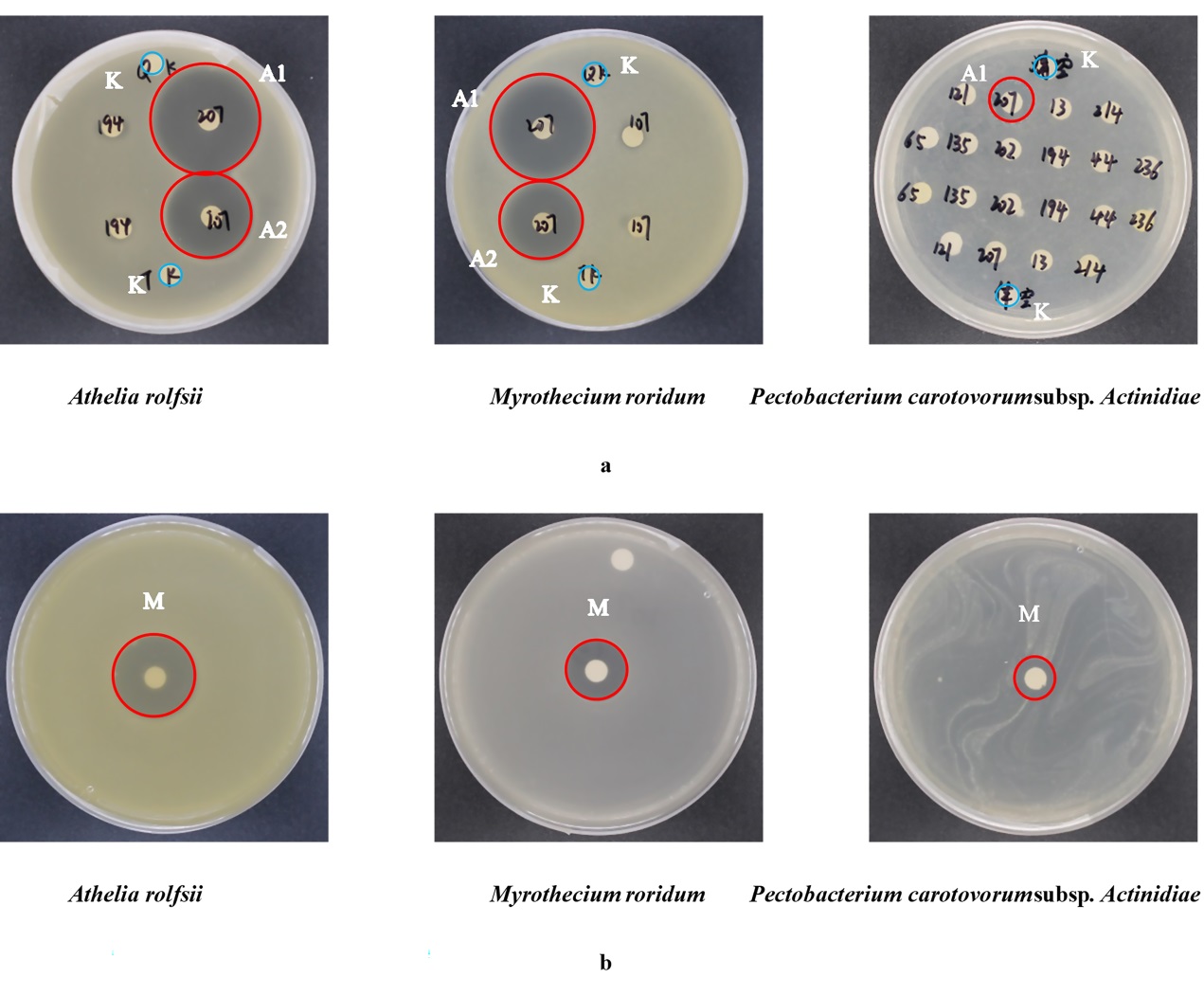
Supplementary Fig.S4- Antimicrobial activity screening of NA-HTong-7 against phytopathogens. (Fig.S4a -Antimicrobial activity of the ethyl acetate extracts of the supernatant and the acetone extracts of the sediment of the NA-HTong-7 against the three phytopathogens; Fig.S4b - Antimicrobial activity of the 70% Mancozeb (750 fold dilution) against the three phytopathogens .A1: the ethyl acetate extracts of the supernatant of the NA-HTong-7; A2: the acetone extracts of the sediment of the HTong-7; K: A negative control; M: 70% Mancozeb (750 fold dilution))**


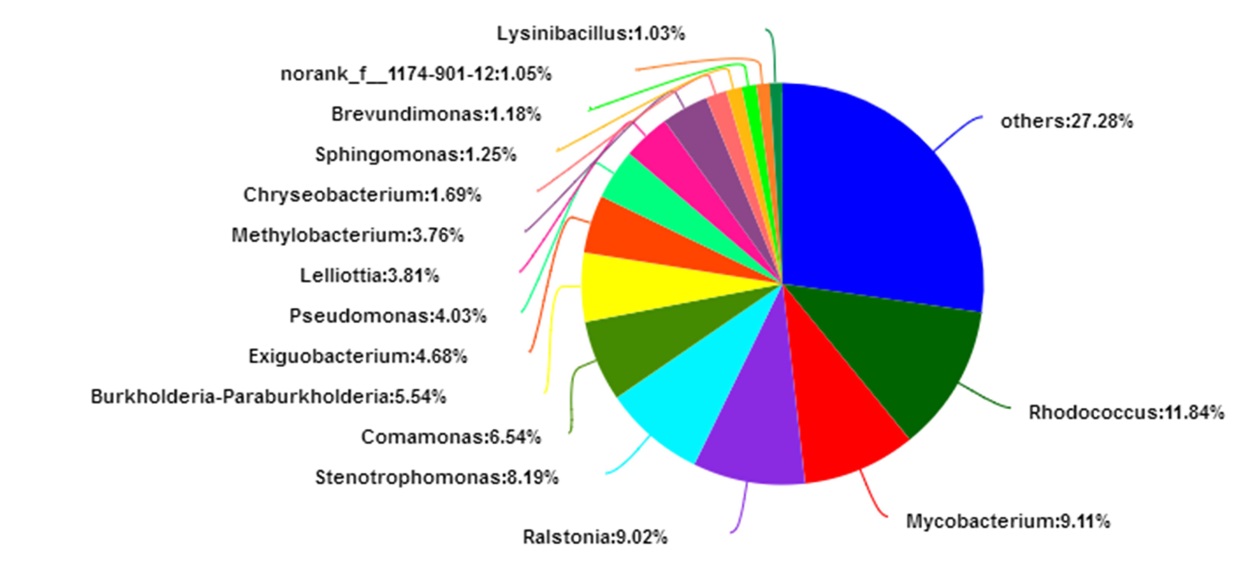


**Supplementary Fig.S5-Community analysis pieplot on Genus level of the culture-independent endophytic bacteria based on Illumina-based analysis. Genera making up less than 1% of total composition in the samples were classified as “other”.**

**
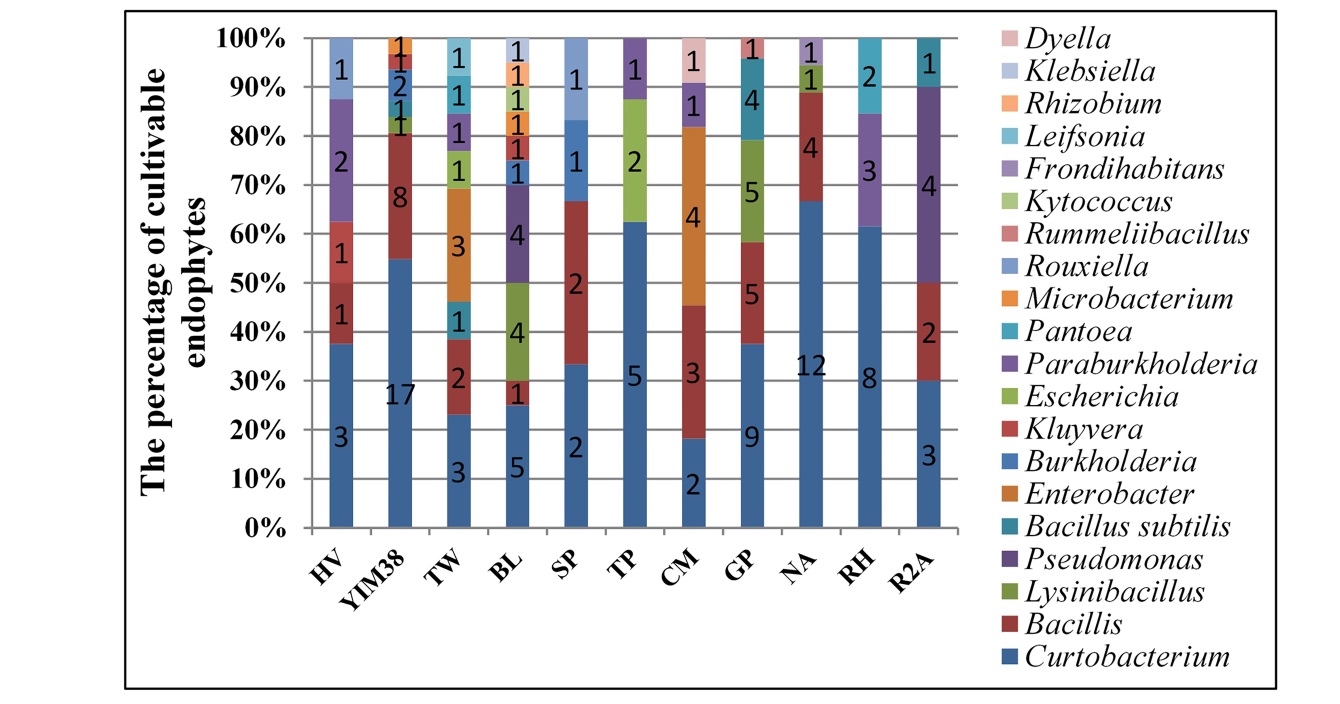
Supplementary Fig.S6- Percentage of cultivable endophytic bacteria screened from the 11 different media (The abscissa represents the 11 different media; The ordinate represents the proportion of different genus, the number of strains of each genus has been indicated in the figure.)**
